# Influenza and respiratory syncytial virus interbreed: Engendering a novel deadly virus

**DOI:** 10.1097/JS9.0000000000000187

**Published:** 2023-01-24

**Authors:** Ruhul Amin, Kuldeep Dhama, Talha B. Emran

**Affiliations:** a Faculty of Pharmaceutical Science, Assam Down Town University, Guwahati, Assam; b Division of Pathology, ICAR-Indian Veterinary Research Institute, Bareilly, Izatnagar, Uttar Pradesh, India; c Department of Pharmacy, BGC Trust University Bangladesh, Chittagong; d Department of Pharmacy, Faculty of Allied Health Sciences, Daffodil International University, Dhaka, Bangladesh

*Dear Editor*,

Highlights

- Viruses from two distinct families merging, bringing their genomes.
- Influenza A particles might hide from the immune system by using the respiratory syncytial virus (RSV) surface proteins.
- RSV often infects deeper into the lung tissue.
- RSV is the most common cause of acute lower respiratory tract infections in infants.

The influenza A virus and the RSV have fused to generate a new hybrid virus that is resistant to the body’s immune response. Viruses from two distinct families merging, bringing their genomes and surface proteins with them. It’s a novel virus that causes disease.

Under a microscope, the gecko’s foot-like hybrid virus is made up of RSV and influenza A virus^1^. The finding was made during a laboratory study of viral interactions during infection, which aimed to provide light on clinical consequences, pathogen behavior, and transmission.

Both viruses, as well as the individual viruses as a control, were introduced to human lung cells. Then, using various types of microscopies, researchers found filamentous features that are indicative of a viral particle that is a hybrid of the two. When these two viruses combine, influenza A seems to infect a wider variety and more human cells^2^. Scientists discovered that influenza A particles might hide from the immune system by using the RSV surface proteins as camouflage^3^.

The hybrid also infected cells without influenza receptors, suggesting that influenza A might potentially enter the lungs and cause a more severe illness if allowed to go further. Since the presence of influenza, A greatly reduces RSV’s potential for reproduction, this union is unfortunately not so terrific for RSV.

Although the experiment was conducted in a controlled laboratory environment, “insufficiently captures the spatial and physiological complexity of the whole respiratory tract. Such brazen borrowing of another virus’s toolkit may play a role in viral pneumonia, as shown by the fact that influenza’s viability is increased when combined with another virus to create a hybrid virus.

Unlike the seasonal flu virus, RSV often infects deeper into the lung tissue, and the farther into the lung it penetrates, the more serious the sickness is likely to be^4^. If we do not take measures to safeguard our health, this (hybridization) is all the more likely to occur, and this is still another reason to avoid becoming infected with several viruses.

RSV is the most common cause of acute lower respiratory tract infections in infants and is often reinfected even years after the first infection. This is despite the fact that influenza A is the most common cause of hospitalization globally^5^.

The results of the research “raise issues about basic laws that control viral assembly,” and it is possible that there are further hybrid viruses waiting to be identified. As with any biological niche, respiratory viruses are part of a larger community of viruses that all infect the same system “Joanne Haney,” a virologist and primary author, made the claim^1^. To have a more complete view of the biology of each virus, we need to know how infections arise in relation to one another.

Having both influenza and RSV proteins allowed the hybrid virus to avoid being neutralized by antibodies and to spread inside infected cells. As several respiratory viruses circulate throughout the winter, the likelihood of comparable recombination processes happening *in vivo* during dual infection is worrisome. Although this only occurred in a laboratory, that fact may provide some comfort. Even nevertheless, the fact that more frequent respiratory viruses are able to take advantage of an evolutionary bottleneck suggests that there will be functional advances of this kind in the wild.

However, the authors believe that coinfections increase the risk of the formation of hybrid virus particles with broad tropism and enhanced immune evasion, even though the formation of hybrid viral particles depends on factors other than structural compatibilities, such as overlapping seasons, geographies, and tropisms.

## Provenance and peer review

Not commissioned, internally peer-reviewed.

## Ethical approval

Not applicable.

## Sources of funding

None.

## Authors’ contribution

R.A.: conceptualization, data curation, writing – original draft preparation, and writing – reviewing and editing. K.D.: data curation, writing – original draft preparation, and writing – reviewing and editing. T.B.E.: writing – reviewing and editing, visualization, and supervision.

## Conflicts of interest disclosure

The authors declare that they have no financial conflict of interest with regard to the content of this report.

## Research registration unique identifying number (UIN)

Not applicable.

## Guarantor

Talha B. Emran.
